# Integrating Tumor Biology and Host Factors in mCRPC: The Prognostic Value of ‘Time to Castration Resistance’, Systemic Inflammation, and Comorbidity Burden in Patients Treated with Enzalutamide

**DOI:** 10.3390/diagnostics16060950

**Published:** 2026-03-23

**Authors:** Seda Sali, Arife Ulaş, Sibel Oyucu Orhan, Sevgi Topçu, Muharrem Koçar, Mürsel Sali, Birol Ocak, Adem Deligönül, Türkkan Evrensel, Erdem Çubukçu

**Affiliations:** 1 Department of Medical Oncology, University of Health Sciences, Bursa City Education and Research Hospital, Bursa 16250, Türkiye; drarifeulas@hotmail.com (A.U.); sibeloyucu@yahoo.com (S.O.O.); muharremkocar@hotmail.com (M.K.); 2 Department of Medical Oncology, Tepecik Training and Research Hospital, İzmir 35120, Türkiye; dr.sevgit@gmail.com; 3 Department of Medical Oncology, School of Medicine, Bursa Uludağ University, Bursa 16059, Türkiye; murselsali@uludag.edu.tr (M.S.); ademd@uludag.edu.tr (A.D.); evrensel@uludag.edu.tr (T.E.); erdemcubukcu@uludag.edu.tr (E.Ç.); 4 Department of Medical Oncology, Bursa Yüksek İhtisas Training and Research Hospital, Bursa 16310, Türkiye; birol08ocak@gmail.com

**Keywords:** metastatic castration-resistant prostate cancer, enzalutamide, systemic immune-inflammation index, time to castration resistance, Charlson comorbidity index, real-world studies

## Abstract

**Background:** Outcomes with enzalutamide in metastatic castration-resistant prostate cancer (mCRPC) are influenced by tumor burden, disease kinetics, and host factors. We evaluated the relative prognostic impact of metastatic pattern, laboratory markers, and prostate-specific antigen (PSA) dynamics in a real-world cohort. **Methods:** We retrospectively analyzed 72 patients with mCRPC treated with enzalutamide. Progression-Free Survival (PFS) and Overall Survival (OS) were estimated using the Kaplan–Meier method. Multivariate Cox proportional hazards models were utilized to identify independent predictors of survival, incorporating clinical variables (visceral metastases, bone tumor burden), kinetic parameters (Time to Castration Resistance [TTCR], Time to PSA Nadir [TTN]), and host factors (Charlson Comorbidity Index [CCI], Eastern Cooperative Oncology Group Performance Status (ECOG PS), Systemic Immune-Inflammation Index [SII], HALP score). **Results:** Visceral metastasis was a dominant predictor of poor outcomes, increasing the risk of death by 4.0-fold (HR: 4.05; 95% CI: 1.84–8.89; *p* < 0.001). A high skeletal tumor burden (≥5 bone lesions) was identified as a critical threshold, associated with a 5.5-fold increase in mortality risk (HR: 5.53; *p* < 0.001). Delays in initiating enzalutamide significantly compromised survival, with each 1-month delay increasing the risk of death by 7.3% (HR: 1.07; *p* = 0.003). While early PSA decline (≥50% at 3 months) did not independently predict OS, a prolonged TTN (>12 months) was associated with superior survival. Notably, host-related factors, including age, CCI, and ECOG PS, were not found to be significantly associated with survival outcomes in this specific dataset. **Conclusions:** Our preliminary findings suggest that survival in real-world mCRPC patients treated with enzalutamide may be influenced predominantly by intrinsic tumor biology—specifically anatomical extent and resistance kinetics—rather than host frailty or comorbidity burden. However, given the retrospective and single-center nature of this study, these findings should be considered hypothesis-generating and require validation in larger, multi-center cohorts. Host-related variables (including age and CCI) were evaluated but were not retained as independent predictors in the final multivariable model. Early initiation of therapy and monitoring of kinetic markers like TTN and TTCR offer superior prognostic stratification compared to static baseline characteristics.

## 1. Introduction

Metastatic castration-resistant prostate cancer (mCRPC) continues to pose a significant global health challenge, remaining a leading cause of cancer-associated mortality despite therapeutic advancements [1]. The clinical management of mCRPC has been transformed by the advent of novel androgen receptor signaling inhibitors (ARSIs). Among these, Enzalutamide serves as a potent, second-generation ARSI that targets the androgen signaling axis through a triple mechanism: it competitively blocks androgen–receptor binding, inhibits nuclear translocation of the receptor complex, and prevents its interaction with Deoxyribonucleic Acid (DNA) [2]. Following the success of pivotal trials such as AFFIRM and PREVAIL, which demonstrated substantial benefits in overall survival (OS) and radiographic progression-free survival (rPFS), Enzalutamide has been established as a standard-of-care regimen in both chemotherapy-naïve and post-docetaxel settings [3,4]. Nevertheless, mCRPC is characterized by marked heterogeneity, and clinical outcomes remain highly variable.

Although Enzalutamide provides survival advantages, the depth and duration of response differ widely among individuals. While some patients achieve long-term disease control, a subset exhibits primary resistance or rapid progression within the initial months of treatment [5,6]. In routine clinical practice, distinguishing patients who are most likely to benefit from ARSIs is essential to optimize treatment sequencing and prevent unnecessary exposure to ineffective therapies. Despite the existence of various prognostic models, there is an unmet need for accessible, cost-effective, and reliable biomarkers that simultaneously reflect tumor biology and the physiological condition of the host.

The prognosis of mCRPC is dictated by a complex interplay between tumor aggressiveness and host-related factors. The duration of sensitivity to initial androgen deprivation therapy (ADT)—referred to as “time to CRPC”—is considered a surrogate marker for the biological behavior of the tumor [5]. Consequently, this metric may offer valuable prognostic insights and aid in patient stratification within real-world cohorts. Conversely, systemic inflammation and host immunity are critical drivers of cancer progression. Recently, inflammatory indices calculated from routine laboratory parameters, such as the Systemic Immune-Inflammation Index (SII) and the Hemoglobin–Albumin–Lymphocyte–Platelet (HALP) score, have gained attention as promising prognostic indicators [7,8]. Additionally, given that the mCRPC population is predominantly elderly, the burden of comorbidities—assessed by the Charlson Comorbidity Index (CCI)—can profoundly influence treatment tolerability and survival [9], a factor often underrepresented in highly selected clinical trial cohorts.

Currently, real-world data evaluating the combined prognostic impact of these clinical, inflammatory, and comorbidity-related factors are scarce. Therefore, this study aimed to investigate the prognostic significance of “Time to CRPC (TTCR),” systemic inflammatory biomarkers (SII, HALP), and comorbidity burden in a real-world cohort of patients with mCRPC treated with Enzalutamide. By integrating these diverse parameters, we sought to identify independent predictors of survival to support more personalized therapeutic decision-making.

## 2. Materials and Methods

This study was designed as a retrospective, single-center analysis. The study protocol was approved by the Bursa City Hospital Ethics Committee (Approval No: 2025-18/9, dated 17 September 2025) covering the review of medical records for all patients treated with Enzalutamide for prostate cancer between January 2018 and July 2025.

A total of 140 patients aged ≥18 years who received Enzalutamide therapy were initially screened. Since the prognosis and treatment landscape differ significantly between hormone-sensitive and castration-resistant disease, the cohort was stratified into two distinct groups. The present study exclusively analyzes the data of 72 patients with mCRPC. The remaining cohort of hormone-sensitive patients was reserved for a separate analysis.

We extracted demographic, pathological, and clinical data from electronic health records. Disease volume and metastatic extent were assessed at baseline using high-sensitivity imaging modalities. Notably, Prostate-Specific Membrane Antigen Positron Emission Tomography/Computed Tomography (PSMA-PET/CT) was utilized in the entire cohort for primary staging and assessment of metastatic burden. This was supplemented by conventional imaging, including Computed Tomography (CT) and Technetium-99m bone scintigraphy, where clinically indicated. Hematological parameters (neutrophil, lymphocyte, platelet counts, hemoglobin, and albumin) were obtained from blood samples collected within two weeks prior to Enzalutamide initiation. The indices were calculated using standard formulas as follows: The SII was calculated as (Platelet count × Neutrophil count)/Lymphocyte count. The HALP score was calculated as (hemoglobin × albumin × absolute lymphocyte count/platelet count) × 10. We calculated the CCI by reviewing each patient’s history of co-existing conditions. Age was included as a weighted factor. Crucially, to preserve the discriminatory power of the index, the primary diagnosis of Prostate Cancer was excluded from the calculation. However, any other concurrent malignancies (e.g., synchronous rectal carcinoma) were retained and scored according to the standard CCI algorithm. The biochemical response was characterized using two parameters: baseline total PSA and the time to PSA nadir (TTN). The time to PSA nadir was defined as the interval from enzalutamide initiation to the documentation of the nadir PSA, representing the lowest absolute value achieved for each individual patient during the course of therapy. For patients demonstrating a sustained PSA decline at the time of their final assessment within the study window, the TTN was recorded as 12 months, representing the minimum observed duration to nadir. To maintain the robustness of the multivariable Cox proportional hazards models and prevent selection bias, baseline total PSA was utilized as the primary biochemical predictor. TTN was assessed exclusively in univariate analyses; its exclusion from the multivariable models was a deliberate methodological choice to ensure the inclusion of ‘primary progressors’ (patients with no documented PSA decline) and those with intermittent missing longitudinal data, thereby preserving the total sample size (*n* = 72) and avoiding the loss of statistical power associated with listwise deletion. We defined TTCR as the duration from the start of ADT for metastatic disease (or date of metastasis) until the confirmation of castration resistance.

Disease progression was defined based on biochemical criteria, radiological evidence, or a combination of both. Detailed information regarding the detection of progression and the precise date of onset was meticulously extracted from the patients’ longitudinal medical records, including physician anamnesis notes and clinical follow-up reports. This comprehensive review of clinical documentation ensured the accurate determination of event dates for survival and progression analyses. Progression-Free Survival (PFS) was calculated from the initiation of Enzalutamide to the date of disease progression (radiographic or clinical) or death from any cause. Patients who did not experience progression or death were censored at the data cut-off date. Overall Survival (OS) was defined as the time interval from the initiation of Enzalutamide to death from any cause. Patients who were still alive at the time of analysis were censored at the data cut-off date. The median follow-up duration was estimated using the reverse Kaplan–Meier method. The study endpoint for censored observations was defined as the data cut-off date of 24 December 2025, or the date of the last documented clinical contact for patients who were lost to follow-up prior to this date.

## 3. Statistical Analysis

Survival analyses were performed using the Kaplan–Meier method, and survival distributions were compared with the log-rank test. Survival outcomes were reported as mean and median survival times along with their standard errors. Variables associated with survival were evaluated using a stepwise backward Cox proportional hazards regression model (Likelihood Ratio method). A two-sided type I error rate of α = 0.05 was considered statistically significant. The proportional hazards assumption was verified for all covariates using the Schoenfeld test. All statistical analyses were conducted using SPSS Statistics (version 27; IBM Corp., Armonk, NY, USA).

## 4. Results

The clinicopathological characteristics of the 72 mCRPC patients are detailed in Table 1. Most patients were <75 years old (68.1%) and had an Eastern Cooperative Oncology Group Performance Status (ECOG PS) of 0–1 (54.2%). High-grade disease (Gleason ≥ 8) was present in 52.8%, and 77.8% had de novo metastases. Disease burden was significant, with 68.1% presenting with ≥5 bone metastases and 16.7% with visceral involvement (M1c). Over half of the cohort (55.6%) received prior docetaxel, while 8.3% had prior abiraterone exposure. Regarding biochemical response, 65.3% of patients achieved a ≥50% PSA decline within 3 months of enzalutamide initiation. Patients were stratified into two groups based on the TTCR, using a median cutoff of 15.4 months: Late Transition (≥15.4 months) and Early Transition (<15.4 months).

After a median follow-up of 34.2 months, progression occurred in 53 patients (73.6%), and 39 patients (54.2%) experienced a death event, reflecting the advanced disease burden and real-world clinical characteristics of this mCRPC cohort. The median PFS and OS were 15.5 months and 24.4 months, respectively (Table 2, Supplementary Figure S1).

**Figure 1:**
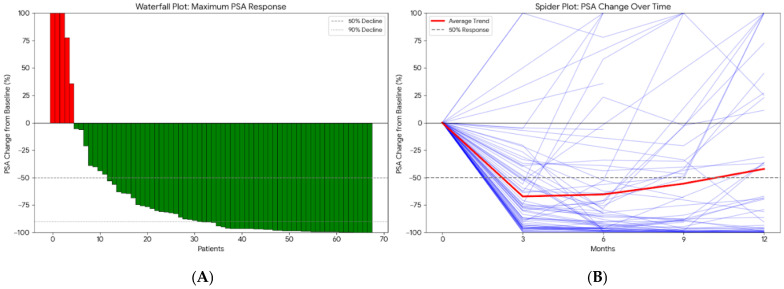
Individual Prostate-Specific Antigen (PSA) (ng/mL) Response and Treatment Kinetics. (**A**) Waterfall plot showing the maximum percentage change in PSA from baseline for each patient (red bars indicate PSA increase, green bars indicate PSA decrease). (**B**) Spider plot illustrating longitudinal PSA changes over time during enzalutamide treatment (purple lines represent individual patient trajectories, and the red line represents the average trend).

Treatment efficacy was evaluated through individual biochemical response and kinetic patterns. The waterfall plot (Figure 1A) demonstrates the best percentage change in PSA levels from baseline for each patient. A significant proportion of the cohort achieved meaningful biochemical responses, with a majority exhibiting a PSA decline of ≥50%. The spider plot (Figure 1B) further illustrates the longitudinal PSA kinetics during enzalutamide therapy. While most patients displayed a rapid and sustained reduction in PSA levels, the plot highlights a subset of patients with early biochemical progression, characterized by a transient initial decline followed by a rapid rise in PSA titers, reflecting the heterogeneity of treatment response in the mCRPC setting (Figure 1).

**Figure 2:**
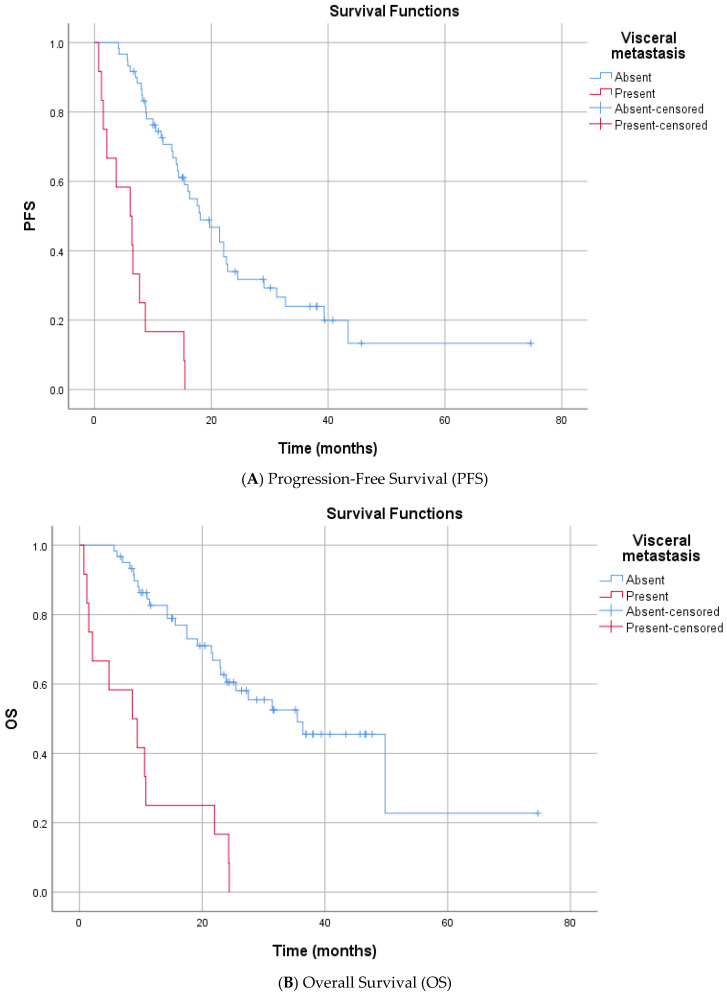
Kaplan–Meier Survival Curves Stratified by Visceral Metastasis.

**Figure 3:**
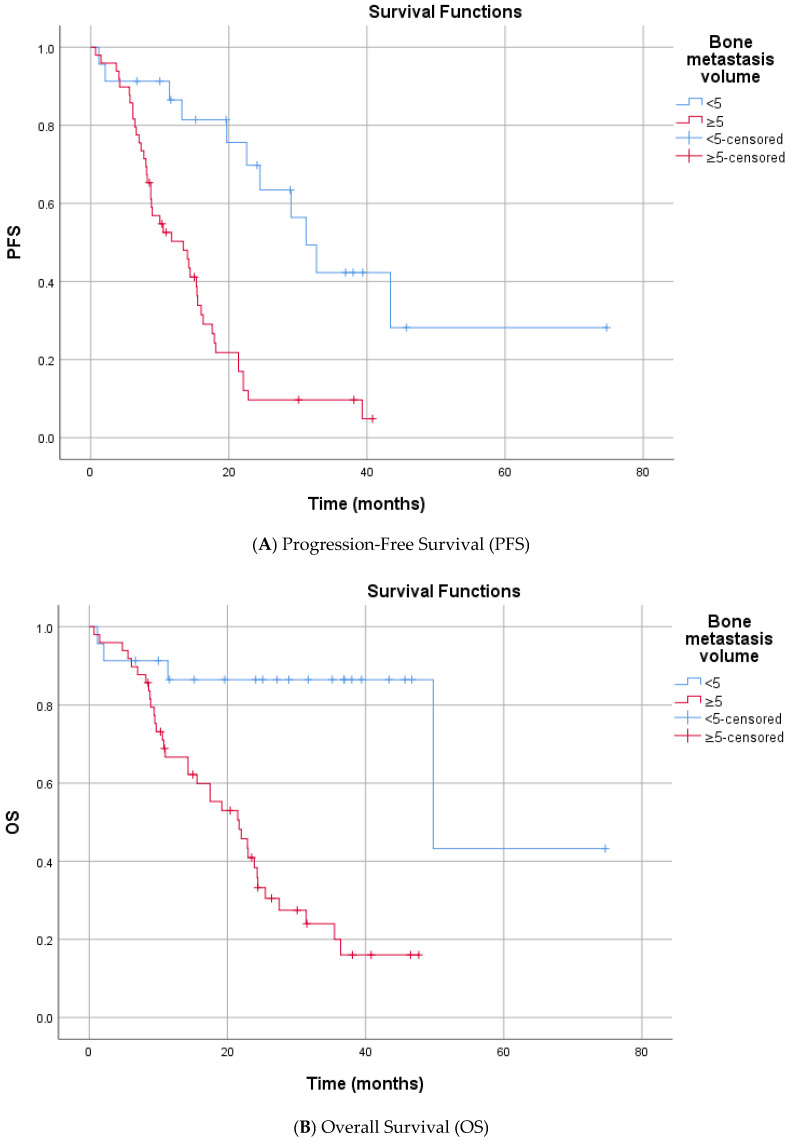
Kaplan–Meier Survival Curves Stratified by Bone Metastasis Volume.

In the univariate analysis (Log-rank test), no significant differences in PFS were observed based on the following variables: age at enzalutamide initiation, ISUP grade group, Gleason category, de novo metastasis status, prior docetaxel therapy, curative therapy history, presence of bone metastasis, surgical history, radiotherapy status, PSA response at 3 months, median HALP score, median SII level, smoking history, alcohol consumption, ECOG PS, and CCI (score < 4 or ≥4) (Supplementary Table S1). In contrast, several factors were significantly associated with shorter PFS. Patients who received Enzalutamide at the first line mCRPC had a significantly longer median PFS compared to the subsequent group (16.3 vs. 8.2 months, *p* = 0.025). The presence of visceral metastasis was associated with a marked reduction in PFS (median 6.1 vs. 18.1 months, *p* < 0.001) (Figure 2). Similarly, patients with high bone metastasis volume (≥5) had a lower median PFS than those with low-volume disease (13.4 vs. 31.2 months, *p* < 0.001) (Figure 3). Metastatic stage was significantly associated with progression-free survival (PFS) (log-rank *p* < 0.001). The median PFS was 43.4 months in patients with M1a disease (*n* = 4), 17.6 months (95% CI, 12.1–23.1) in the M1b group (*n* = 56), and 6.1 months (95% CI, 1.5–10.7) in the M1c group (*n* = 12). Pairwise comparisons demonstrated significantly shorter PFS in M1c compared with M1a (*p* = 0.002) and M1b (*p* < 0.001), whereas no significant difference was observed between M1a and M1b (*p* = 0.180). Furthermore, prior abiraterone use was a significant predictor of resistance, with pre-treated patients exhibiting significantly shorter PFS than ARSI-naïve patients (median PFS: 5.6 vs. 16.3 months; *p* < 0.001). Among biochemical markers, elevated Lactate Dehydrogenase (LDH) (median 6.6 vs. 17.9 months, *p* < 0.001) and elevated Alkaline Phosphatase (ALP) (median 8.7 vs. 21.4 months, *p* < 0.001) were associated with rapid progression. Progression-free survival differed significantly according to the TTN (*p* < 0.001). The median PFS was 8.7 months for patients reaching PSA nadir at 3 months, 16.0 months at 6 months, and 13.2 months at 9 months. Patients with a PSA nadir at ≥12 months had a median PFS of 43.4 months. Pairwise comparisons showed significant differences between 3 vs. 6 months (*p* = 0.049), 3 vs. ≥12 months (*p* < 0.001), 6 vs. ≥12 months (*p* < 0.001), and 9 vs. ≥12 months (*p* < 0.001), whereas no significant differences were observed for 3 vs. 9 months (*p* = 0.195) or 6 vs. 9 months (*p* = 0.776) (Supplementary Table S1, Supplementary Figure S2).

Univariate analysis for OS indicated that age, smoking, alcohol, and ECOG PS did not significantly impact mortality risk. Similar prognostic trends were observed for OS. First enzalutamide initiation at the mCRPC was associated with significantly extended survival compared to delayed treatment (median 31.4 vs. 9.7 months, *p* < 0.001). Significant predictors of increased mortality risk included the presence of visceral metastases (median 8.6 vs. 35.5 months, *p* < 0.001) and high bone metastasis volume (median 21.7 vs. 49.8 months, *p* < 0.001) (Figure 2 and Figure 3). Metastatic stage was significantly associated with OS (*p* < 0.001). The median OS was 49.8 months in patients with M1a disease (*n* = 4), 31.4 months (95% CI, 19.5–43.3) in the M1b group (*n* = 56), and 8.6 months (95% CI, 0.8–16.4) in the M1c group (*n* = 12). Pairwise comparisons demonstrated significantly shorter OS in patients with M1c disease compared with both M1a (*p* = 0.002) and M1b (*p* < 0.001). No statistically significant difference in OS was observed between the M1a and M1b groups (*p* = 0.219). Prior abiraterone exposure was significantly associated with OS (*p* = 0.005). The median OS was 25.5 months (95% CI, 16.4–34.6) in abiraterone-naïve patients (*n* = 66), compared with 5.6 months (95% CI, 4.0–7.2) in patients with prior abiraterone use (*n* = 6). Regarding laboratory parameters, elevated ALP (median 14.3 vs. 49.8 months, *p* < 0.001) and elevated LDH (median 10.8 vs. 27.5 months *p* = 0.050) were all associated with inferior OS. A lower HALP score was significantly associated with poorer OS. The median OS was 21.7 months in patients with low HALP scores, while the median OS was not reached (NR) in those with high HALP scores (*p* = 0.015), indicating prolonged survival in the latter group. Overall survival differed significantly according to the TTN (*p* < 0.001). The median OS was 15.6 months in patients who reached PSA nadir at 3 months (*n* = 15), 25.5 months at 6 months (*n* = 16), 24.4 months at 9 months (*n* = 6), and 49.8 months in those reaching PSA nadir at ≥12 months (*n* = 15). Pairwise comparisons demonstrated significantly longer OS for patients with a PSA nadir at ≥12 months compared with those at 3 months (*p* < 0.001), 6 months (*p* < 0.001), and 9 months (*p* < 0.001). No statistically significant differences in OS were observed between 3 vs. 6 months (*p* = 0.121), 3 vs. 9 months (*p* = 0.094), or 6 vs. 9 months (*p* = 0.330) (Supplementary Table S1, Supplementary Figure S2).

In multivariable analyses, Cox proportional hazards regression models were constructed separately for PFS and OS. Variables with a *p* value ≤ 0.05 in univariate analyses for PFS and OS were considered eligible for inclusion in the multivariable models. In addition, age, CCI, baseline total PSA level, TTCR, SII, and HALP score were predefined and entered into the models as continuous variables for both PFS and OS, irrespective of their univariate significance. A stepwise backward selection method (Likelihood Ratio) was applied to derive the final multivariable models, and only statistically significant variables retained in the final models were considered for interpretation. Although TTN was significantly associated with both PFS and OS in univariate analyses, this variable was not included in the multivariable models due to incomplete follow-up data, which substantially reduced the effective sample size when incorporated. To preserve statistical power and model stability, baseline total PSA was therefore included as a continuous variable in place of PSA nadir timing (Supplementary Table S2).

In the multivariable Cox proportional hazards regression analysis, several factors were identified as independent predictors of shorter PFS (overall model *p* < 0.001). The presence of visceral metastases was associated with a markedly increased risk of disease progression, with patients harboring visceral metastases having a 4.22-fold higher risk of PFS events compared with those without visceral involvement (HR 4.22; 95% CI, 1.91–9.29; *p* < 0.001). Similarly, patients with ≥5 bone metastases exhibited a significantly increased risk of progression compared with those with fewer than five bone lesions (HR 2.95; 95% CI, 1.40–6.20; *p* = 0.004). A longer interval between the development of mCRPC and the initiation of enzalutamide treatment was independently associated with inferior progression-free survival, with each additional month of delay conferring a 6.3% increase in the risk of progression (HR 1.06; 95% CI, 1.02–1.10; *p* = 0.002). Elevated lactate dehydrogenase (LDH) levels were similarly associated with poorer outcomes; patients with LDH ≥225 U/L had a 2.07-fold higher risk of progression compared with those with lower LDH levels (HR 2.07; 95% CI, 1.06–4.04; *p* = 0.034). In addition, a higher SII remained an independent prognostic factor for PFS, with each unit increase in SII conferring a modest but statistically significant increase in progression risk (HR 1.06; 95% CI, 1.020–1.105; *p* = 0.011) (Table 3, Supplementary Figure S3).

In the multivariable Cox regression analysis, several variables were independently associated with OS (overall model *p* < 0.001). The presence of visceral metastases was associated with a significantly increased risk of death (HR 4.05; 95% CI, 1.87–8.76; *p* < 0.001). Likewise, patients with ≥5 bone metastases had a markedly higher mortality risk compared with those with fewer than five bone lesions (HR 5.53; 95% CI, 1.64–18.66; *p* = 0.006). A shorter interval to the development of castration resistance was independently associated with inferior OS. Specifically, each one-month decrease in the time to mCRPC was associated with a 2.1% increase in the risk of death (HR 0.98 per month; 95% CI, 0.96–1.00; *p* = 0.037). In addition, the time interval between the diagnosis of mCRPC and initiation of enzalutamide was included in the model as a continuous variable. A longer delay in starting enzalutamide was associated with worse overall survival, with each additional month of delay increasing the risk of death by 7.3% (HR 1.07; 95% CI, 1.03–1.12; *p* < 0.001). Higher baseline total PSA levels were also significantly associated with increased mortality risk, with each unit increase corresponding to a 0.3% rise in the risk of death (HR 1.003; 95% CI, 1.001–1.004; *p* < 0.001) (Table 4, Supplementary Figure S3).

## 5. Discussion

The clinical management of mCRPC necessitates a multi-dimensional approach that integrates tumor biology, disease burden, and host-related factors. The primary objective of this investigation was to elucidate the complex prognostic interplay between host-related factors—quantified via the CCI and systemic inflammatory-nutritional indices (SII and HALP)—and intrinsic tumor biology, as reflected by the TTCR. Furthermore, we interrogated the prognostic significance of biochemical kinetics, specifically baseline PSA levels and the velocity of PSA decline toward an individualized nadir, within a multifaceted framework to identify independent predictors of survival in a real-world mCRPC cohort receiving enzalutamide. By integrating these heterogeneous clinical, biological, and host-dependent parameters, this study provides a granular perspective on the determinants of treatment durability and overall survival.

In the AFFIRM trial, which included mCRPC patients previously treated with chemotherapy, the median OS was reported as 18.4 months, whereas the PREVAIL trial, enrolling chemotherapy-naïve patients, demonstrated a median OS of 36 months [3,4]. In our cohort, the median OS was 24.4 months. Given that 55.6% of patients in our study had received prior docetaxel chemotherapy, the observed survival outcomes appear comparable to and in line with previously published data, supporting the external validity of our real-world findings.

When patient characteristics were evaluated, age distribution differed modestly across studies. In the AFFIRM trial, 69.8% of patients were aged 65 years or older, whereas in the PREVAIL study, 36.4% of patients were 75 years or older [3,4]. In our real-world cohort, 31.9% of patients were aged 75 years or older, suggesting an age profile broadly comparable to that of landmark trials. In contrast, marked differences were observed with respect to performance status. In PREVAIL, patients with ECOG performance status 2 were excluded, and the majority of patients had an ECOG score of 0. Similarly, in AFFIRM, only approximately 8.5% of patients had an ECOG performance status of 2, with the remaining patients classified as ECOG 0–1 [3,4]. In our cohort, however, a substantially higher proportion of patients (45.8%) had an ECOG performance status ≥ 2, reflecting a less selected and more clinically complex population. In addition, comorbidity burden was systematically assessed in our study using the CCI, which was not routinely incorporated into AFFIRM or PREVAIL. More than half of the patients (56.9%) had a CCI ≥ 4, indicating a high comorbidity load. Despite the inclusion of patients with poorer performance status and higher age, CCI, and ECOG performance status were not independently associated with PFS or OS in our analyses, suggesting that enzalutamide may confer clinical benefit even in patients with less favorable baseline characteristics in routine clinical practice.

Our multivariable analyses demonstrated that visceral metastatic involvement (M1c) was a strong and independent predictor of adverse outcomes. Compared with patients with bone- or nodal-only disease, the presence of visceral metastases was associated with a 4.2-fold increased risk of radiographic progression (PFS HR: 4.22) and a 4.0-fold higher risk of death (OS HR: 4.05). These findings are consistent with observations from the PREVAIL trial, in which patients with visceral disease—particularly liver metastases—derived substantially less survival benefit from enzalutamide than those without visceral involvement [4]. Similarly, in the AFFIRM trial, although enzalutamide demonstrated a survival advantage over placebo in the overall population, the magnitude of benefit was attenuated among patients with visceral metastases [3]. As discussed by Zheng et al. [6], visceral involvement has been associated with lineage plasticity, neuroendocrine differentiation, and androgen receptor-independent disease states, which may limit the durability of AR-targeted therapies.

Regarding skeletal tumor burden, we identified five or more bone metastases as a clinically meaningful threshold associated with adverse outcomes. Patients exceeding this cutoff experienced a 2.9-fold increased risk of progression (PFS HR: 2.95) and a 5.5-fold higher risk of death (OS HR: 5.53). These findings indicate that an increased number of bone metastases remains a powerful prognostic determinant in the mCRPC setting. While higher cutoffs for bone metastatic burden have been used in clinical trials to stratify risk [3,4]; our real-world data suggest that a lower threshold may be sufficient to identify a particularly high-risk subgroup in routine practice. This observation is likely influenced by the inclusion of a less selected population, characterized by a substantial proportion of patients with impaired performance status (ECOG ≥ 2 in 45.8%).

In our cohort, initiating enzalutamide in the first-line mCRPC setting was associated with longer PFS and OS compared with its use in later lines. When the interval from mCRPC onset to enzalutamide initiation was modeled as a continuous variable, each 1-month delay was associated with a statistically significant increase in both mortality risk (OS: +7.3% per month; HR 1.07) and progression risk (PFS: +6.3% per month; HR 1.06). This observation is biologically and clinically plausible, and is supported by the broader evidence that outcomes with enzalutamide are generally more favorable when the drug is used earlier in the treatment sequence—most notably in the chemotherapy-naïve population in PREVAIL compared with the post-chemotherapy population in AFFIRM, reflecting differences in disease biology, prior treatment exposure, and performance status at treatment initiation [3,4,10]. However, this observation should be interpreted cautiously. In real-world clinical practice, treatment sequencing is influenced by disease burden, prior therapies, physician preference, and patient condition. Therefore, a longer mCRPC–enzalutamide interval may partly reflect underlying disease aggressiveness or prior systemic treatment exposure rather than a direct detrimental effect of delayed enzalutamide initiation. Although our multivariable models adjusted for key tumor-related factors, including visceral metastases, bone disease burden, LDH, and baseline PSA, residual confounding cannot be excluded given the retrospective design. Accordingly, this finding should be considered hypothesis-generating rather than evidence of a causal relationship.

These findings are in line with the report by Nadal et al., where enzalutamide showed higher PSA responses and longer progression metrics in docetaxel-naïve patients; however, after adjustment for baseline disease characteristics, prior docetaxel exposure did not remain an independent determinant of overall survival [11]. Prior systemic therapy exposure, including docetaxel and abiraterone, was evaluated separately in the present study. However, due to the limited number of patients (*n* = 4) receiving multiple prior lines, detailed stratification according to complex sequencing patterns was not feasible.

Importantly, the variables that retained prognostic significance in that study—such as ECOG performance status, visceral metastases, and alkaline phosphatase—closely parallel the independent factors identified in our multivariable models. Taken together, these observations indicate that prior docetaxel exposure was not associated with survival differences in our cohort, even at the univariate level, and reinforce the notion that outcomes under enzalutamide are predominantly determined by underlying disease burden and patient-related factors rather than chemotherapy history alone.

In our cohort, patients with prior abiraterone exposure exhibited shorter PFS and OS in univariate analyses; however, this association did not persist in multivariable models, likely due to the limited number of abiraterone-pretreated patients. This observation can be interpreted within the context of existing literature. Azad et al. reported that molecular alterations detectable in circulating cell-free DNA may be associated with therapeutic resistance in mCRPC, supporting the possibility that prior AR-targeted therapy could influence subsequent treatment responses [12]. Nevertheless, clinical data from a randomized crossover trial evaluating the sequencing of abiraterone and enzalutamide demonstrated that enzalutamide retains meaningful activity when used after abiraterone, whereas the reverse sequence appears less effective [13]. Taken together, these findings suggest that although prior abiraterone exposure may be associated with attenuated responses in some patients, enzalutamide can still provide clinical benefit in this setting, which is consistent with the absence of independent prognostic significance in our multivariable analysis.

In our study, TTCR was initially examined in univariate analyses using the cohort median as a categorical threshold (median 15.4 months). When evaluated in this manner, TTCR did not demonstrate a significant association with either PFS or OS. However, given its recognized clinical relevance, TTCR was subsequently incorporated into the multivariable Cox models as a continuous variable. In this analysis, longer TTCR was independently associated with improved overall survival, indicating that the duration of response to initial androgen deprivation remains an important prognostic marker when assessed on a continuous scale. This finding is in line with prior reports that have used similar cut-off values (e.g., approximately 16 months) and demonstrated that prolonged TTCR, particularly when accompanied by favorable laboratory indices such as higher HALP scores, correlates with improved survival outcomes [8]. Furthermore, studies conducted in metastatic castration-sensitive populations have also shown that a longer interval to castration resistance is associated with more favorable long-term prognosis [5]. Together, these data support the concept that TTCR reflects underlying tumor biology and remains a meaningful prognostic indicator across different stages of advanced prostate cancer.

In our cohort, early PSA decline kinetics (specifically a ≥50% reduction at 3 months) did not independently predict OS. In contrast, TTN emerged as a more informative prognostic indicator. We observed that a TTN exceeding 12 months was associated with superior survival, a finding that is consistent with prior reports, including the analysis by Sasaki et al., which demonstrated an inverse relationship between TTN and disease progression in advanced prostate cancer [14]. Due to incomplete longitudinal PSA data, serial PSA measurements could not be incorporated into the multivariable models. Instead, baseline PSA was entered into the Cox regression analyses as a continuous variable. While baseline PSA was not significantly associated with PFS, higher initial PSA levels were independently associated with inferior OS (HR 1.003 per unit increase).

When additional laboratory parameters were evaluated, elevated ALP and LDH levels were significantly associated with shorter PFS and OS in univariate analyses. In contrast, the median value of SII was not associated with differences in either survival endpoint. Similarly, while the median HALP value did not differentiate patients in terms of PFS, higher HALP values were associated with improved OS at the univariate level. In multivariable Cox regression analyses—where HALP and SII were entered as continuous variables—only elevated LDH and higher SII remained independently associated with shorter PFS, whereas none of these laboratory parameters retained independent significance for OS. The literature addressing the prognostic value of SII in advanced prostate cancer has yielded heterogeneous results. Several studies have identified low SII as a marker of favorable survival, while others, similar to our findings, have reported no independent association with long-term outcomes after adjustment for clinical variables [7,15]. Although a growing body of evidence suggests that a low HALP score is strongly indicative of poor prognosis due to the convergence of malnutrition and systemic inflammation [8,16]; our multivariate analysis did not confirm HALP as an independent predictor of survival. This discrepancy likely suggests that in our specific real-world cohort, the dominant impact of heavy anatomical tumor burden (e.g., visceral metastases and high bone volume) may have overshadowed the subtle prognostic contributions of these inflammatory-nutritional indices.

In our study, neither ECOG performance status nor CCI demonstrated a significant association with PFS or OS. In univariate analyses, CCI categorized as <4 versus ≥4 did not show a meaningful impact on survival outcomes. To further explore its prognostic relevance, CCI was also incorporated into the multivariable Cox regression models as a continuous variable. However, even in this form, CCI did not retain statistical significance in the stepwise selection process. Notably, this observation is consistent with prior reports in the literature. A meta-analysis evaluating the prognostic impact of CCI in prostate cancer demonstrated that while comorbidity burden was associated with all-cause mortality, it did not independently predict prostate cancer-specific mortality [17]. Similarly, in a study conducted in men with mCRPC treated within a clinical trial setting, CCI was not found to be an independent predictor of overall survival after adjustment for established clinical prognostic factors [18]. Together with our findings, these data suggest that in the advanced mCRPC setting, tumor biology and disease burden may outweigh the prognostic contribution of comorbidity indices in determining survival outcomes under systemic therapy.

While host-related factors, including age, CCI, and ECOG PS, did not reach statistical significance as independent predictors in our multivariable models, these results should be interpreted with caution. In a cohort of this size (*n* = 72) with 39 documented death events, the study may have been underpowered to detect subtle but clinically relevant impacts of host frailty. The relatively wide confidence intervals observed for established predictors, such as visceral and bone metastatic burden, further underscore the limitations in precision inherent to smaller real-world datasets.

## 6. Strengths and Limitations

The primary strength of this investigation lies in its pragmatic, real-world design, which captured a clinically complex patient population often excluded from pivotal randomized controlled trials. By including a substantial proportion of patients with poor performance status (ECOG ≥ 2) and significant comorbidity burdens (CCI ≥ 4), our findings offer high external validity and are directly applicable to routine oncologic practice. Furthermore, the integration of granular kinetic parameters—specifically TTCR and TTN—alongside detailed anatomical tumor burden quantification provides a more dynamic prognostic framework than static baseline assessments alone.

Several limitations of our study must be acknowledged. First, the retrospective, single-center design and the relatively small sample size may limit the generalizability of our findings. Second, our cohort was heterogeneous regarding treatment lines, including both first-line and subsequent-line enzalutamide use. Furthermore, the availability of subsequent therapies, such as radioligand therapy (e.g., Lu-177 PSMA), may have evolved during the study period and could potentially influence overall survival outcomes, a factor that was not fully accounted for in our analysis. In addition, incomplete longitudinal PSA data restricted the incorporation of dynamic PSA variables into multivariable modeling. Finally, radiographic progression assessments were based on routine clinical practice rather than centralized review.

## 7. Conclusions

In conclusion, our findings demonstrate that in patients with mCRPC treated with enzalutamide, anatomical tumor burden—particularly visceral metastases and extensive bone involvement—along with disease kinetics reflected by TTCR, TTN, and time from mCRPC onset to enzalutamide initiation, are the principal determinants of survival. In contrast, age, ECOG PS performance status, and comorbidity burden did not independently influence outcomes in this real-world cohort.

These results suggest that the effectiveness of enzalutamide is driven predominantly by tumor biology and disease extent rather than host frailty, supporting its clinical utility even in less selected patient populations. This multidimensional prognostic perspective may help clinicians better stratify patients and optimize treatment sequencing in routine mCRPC practice.

## Figures and Tables

**Table 1:** Baseline Demographic and Clinical Characteristics of the Study Population (*n* = 72).

Characteristic	Category	*n* (%)
Demographic & General		
Age (years)	<75 years	49 (68.1%)
	≥75 years	23 (31.9%)
ECOG PS	0–1 (Preserved)	39 (54.2%)
	≥2 (Restricted)	33 (45.8%)
Charlson Comorbidity Index	<4 score	31 (43.1%)
	≥4 score	41 (56.9%)
Primary Tumor & Diagnosis		
ISUP Grade Group	2–3	17 (23.6%)
	4	12 (16.7%)
	5	26 (36.1%)
	Unknown	17 (23.6%)
Gleason Category	<8	17 (23.6%)
	≥8	38 (52.8%)
	Unknown	17 (23.6%)
De Novo Metastasis	No	16 (22.2%)
	Yes	56 (77.8%)
Curative Therapy History	Absent	57 (79.2%)
	Present	15 (20.8%)
Prior Prostate Surgery	No	58 (80.6%)
	Yes	14 (19.4%)
Radiotherapy History	None	10 (13.9%)
	Palliative	43 (59.7%)
	Primary	18 (25.0%)
Disease Burden at mCRPC		
TTCR	Late mCRPC Transition (≥15.4 months)	37 (51.4%)
	Early mCRPC Transition (<15.4 months)	35 (48.6%)
Enzalutamide Timing (first mCRPC or subsequent line)	First	62 (86.1%)
	Subsequent	10 (13.9%)
Visceral Metastasis	Absent	60 (83.3%)
	Present	12 (16.7%)
Bone Metastasis	Absent	6 (8.3%)
	Present	66 (91.7%)
Bone Metastasis Volume	<5 metastases	23 (31.9%)
	≥5 metastases	49 (68.1%)
Metastatic Stage	M1a	4 (5.6%)
	M1b	56 (77.8%)
	M1c	12 (16.7%)
Systemic Therapies		
Prior Docetaxel	No	32 (44.4%)
	Yes	40 (55.6%)
Prior Abiraterone	No	66 (91.7%)
	Yes	6 (8.3%)
Bone-Targeted Therapy	No	34 (47.2%)
	Yes	38 (52.8%)
Biochemical Markers		
PSA Response at 3 Months	≥50% decline	47 (65.3%)
	<50% decline	13 (18.1%)
	Unknown	12 (16.7%)
TTN	3–6 months	31 (43.1%)
	≥9 months	21 (29.2%)
	Unknown	20 (27.8%)
LDH Level (U/L)	<225	52 (72.2%)
	≥225	18 (25.0%)
	Unknown	2 (2.8%)
ALP Level (U/L)	<130	44 (61.1%)
	≥130	28 (38.9%)
HALP Score (Median) 31.3 (IQR: 20.8–45.6)	<31	35 (48.6%)
	≥31	37 (51.4%)
SII Level (Median) 654.3 (IQR: 408.6–1053.5)	<654	36 (50.0%)
	≥654	36 (50.0%)

Abbreviations: ALP, alkaline phosphatase (U/L, units per liter); ECOG PS, Eastern Cooperative Oncology Group performance status (dichotomized as 0: original score 0–1; 1: original score ≥ 2); HALP, hemoglobin, albumin, lymphocyte, and platelet index; IQR, Interquartile Range; ISUP, International Society of Urological Pathology; LDH, lactate dehydrogenase (U/L, units per liter); M1a/b/c, TNM metastatic staging; mCRPC, metastatic castration-resistant prostate cancer; *n*, number of patients; PSA, prostate-specific antigen (ng/mL); SII, systemic immune-inflammation index; TTCR, time to metastatic castration-resistant prostate cancer; TTN, time to PSA nadir. Continuous variables are presented as Median (Interquartile Range [IQR]: 25th–75th percentiles).

**Table 2:** Summary of Survival Estimates for the Total Cohort (*n* = 72).

	*n*/Event	Survival Mean	SE	95% CI of Mean	Survival Median	SE	95% CI of Median
OS	72/39	33.73	4.45	25.01–42.45	24.40	3.95	16.65–32.15
PFS	72/53	22.87	2.99	17.00–28.74	15.50	1.79	11.99–19.02

Abbreviations: CI, confidence interval; OS, overall survival; PFS, progression-free survival; SE, standard error.

**Table 3:** Multivariate Cox Proportional Hazards Model for Progression-Free Survival (PFS).

Variable	*p*-Value	HR	95% CI
Visceral Metastasis (Present vs. Absent)	<0.001	4.22	1.914–9.287
Bone Met. Volume (≥5 vs. <5)	0.004	2.95	1.401–6.203
LDH Level (≥225 vs. <225 U/L)	0.034	2.07	1.057–4.039
SII (Score)	0.011	1.06	1.020–1.105 *
mCRPC—Enzalutamide Duration	0.002	1.06	1.022–1.104

Model Significance: *p* < 0.001. Abbreviations: CI, confidence interval; HR, hazard ratio; LDH, lactate dehydrogenase; mCRPC, Metastatic castration-resistant prostate cancer; PSA, prostate-specific antigen; SE, standard error; SII, systemic immune-inflammation index. A stepwise backward (Likelihood Ratio) method was used for model selection. * SII has been rescaled (per 100-unit increase) to ensure a clinically meaningful Hazard Ratio as per reviewer request.

**Table 4:** Multivariate Cox Regression Analysis for Overall Survival (OS).

Variable	*p*-Value	HR	95% CI
Visceral Metastasis (Present vs. Absent)	<0.001	4.05	1.868–8.764
Bone Metastasis Volume (≥5 vs. <5)	0.006	5.53	1.638–18.662
TTCR (months)	0.037	0.98	0.960–0.999
Baseline Total PSA (ng/mL)	<0.001	1.00	1.001–1.004
mCRPC—Enzalutamide Duration	<0.001	1.07	1.033–1.115

Model Significance: *p* < 0.001. Abbreviations: CI, confidence interval; HR, hazard ratio; LDH, lactate dehydrogenase; mCRPC, Metastatic castration-resistant prostate cancer; PSA, prostate-specific antigen; SE, standard error; SII, systemic immune-inflammation index; TTCR, time to metastatic castration-resistant prostate cancer. A stepwise backward (Likelihood Ratio) method was used for model selection.

## Data Availability

The data supporting the findings of this study were obtained from the electronic medical record system of Bursa City Education and Research Hospital and represent routinely collected clinical information. Owing to confidentiality considerations and ethics-related restrictions, the raw data cannot be made publicly available. De-identified datasets may be shared by the corresponding author upon reasonable request, subject to approval by the institutional ethics committee.

## Supplementary Materials

The following supporting information can be downloaded at: https://www.mdpi.com/article/10.3390/diagnostics16060950/s1, Supplementary Table S1. Univariate Analysis (Log-rank) for Overall Survival and Progression-Free Survival Based on Baseline Characteristics (*n* = 72). Supplementary Table S2: Candidate Variables Entered into the Initial Multivariable Cox Regression Models. Supplementary Figure S1. Kaplan-Meier survival curves for patients with metastatic castration-resistant prostate cancer. (A) Progression-free survival (PFS) of the study population. (B) Overall survival (OS) of the study population. Supplementary Figure S2. Kaplan–Meier survival curves according to time to PSA nadir (TTN) categories. Supplementary Figure S3: Forest plot of multivariable Cox proportional hazards models for progression-free survival (PFS) and overall survival (OS).
