# Supplementary material for: Integrating Tumor Biology and Host Factors in mCRPC: The Prognostic Value of ‘Time to Castration Resistance’, Systemic Inflammation, and Comorbidity Burden in Patients Treated with Enzalutamide
Source: Diagnostics (Basel). 2026 Mar 23;16(6):950. doi: 10.3390/diagnostics16060950 (PMC13025191; doi:10.3390/diagnostics16060950)
Supplement: Supplementary file 1 [file diagnostics-16-00950-s001.zip › diagnostics-4159136-supplementary.pdf]

Supplementary Table S1: Univariate Analysis (Log-rank) for Overall Survival and Progression-Free Survival Based on Baseline Characteristics (n = 72)

**Variable & Subgroups**	**n (%)**	**OS (p-value)**	**PFS (p-value)**
**Demographic and Clinical**			
Age at Enzalutamide Start			
< 75 years	49 (68.1%)	0.646	0.385
≥ 75 years	23 (31.9%)		
ECOG Performance Status			
0	39 (54.2%)	0.121	0.203
1	33 (45.8%)		
CCI			
< 4 score	31 (43.1%)	0.632	0.798
≥ 4 score	41 (56.9%)		
Smoking History			
No	24 (33.3%)	0.630	0.859
Yes	30 (41.7%)		
Unknown	18 (25.0%)		
Alcohol Consumption			
No	5 (6.9%)	0.151	0.223
Yes	43 (59.7%)		
Unknown	24 (33.3%)		
**Tumor Characteristics**			
Grade Category (Gleason)			
< 8	17 (23.6%)	0.075	0.079
≥ 8	38 (52.8%)		

**Variable & Subgroups**	**n (%)**	**OS (p-value)**	**PFS (p-value)**
De Novo Metastasis			
No	16 (22.2%)	0.078	0.076
Yes	56 (77.8%)		
**Disease Burden and Metastasis**			
TTCR Median			
Late mCRPC Transition (≥15.4 months)	37 (51.4%)	0.526	0.748
Early mCRPC Transition (<15.4 months)	35 (48.6%)		
Enzalutamide Timing (first mCRPC or subsequent line)			
First	62 (86.1%)	**<0.001**	**0.025**
Subsequent	10 (13.9%)		
Visceral Metastasis			
Absent	60 (83.3%)	**<0.001**	**<0.001**
Present	12 (16.7%)		
Bone Metastasis			
Absent	6 (8.3%)	0.683	0.500
Present	66 (91.7%)		
Bone Metastasis Volume			
< 5 metastases	23 (31.9%)	**<0.001**	**<0.001**
≥ 5 metastases	49 (68.1%)		
Metastatic Stage			
M1a	4 (5.6%)		**<0.001**

**Variable & Subgroups**	**n (%)**	**OS (p-value)**	**PFS (p-value)**
M1b	56 (77.8%)	**<0.001**<br>a-b: 0,219<br>**a-c:0,002**<br>**b-c:<0,001**	a-b:0,180<br>**a-c:0,002**<br>**b-c:<0,001**
M1c	12 (16.7%)		
**Treatment History**			
Prior Docetaxel Therapy			
No	32 (44.4%)	0.589	0.159
Yes	40 (55.6%)		
Prior Abiraterone Use			
No	66 (91.7%)	**0.005**	**<0.001**
Yes	6 (8.3%)		
Prior Curative Treatment			
No	57 (79.2%)	0.062	0.080
Yes	15 (20.8%)		
Prior Prostate Surgery			
No	58 (80.6%)	0.803	0.750
Yes	14 (19.4%)		
Radiotherapy History			
None	10 (13.9%)	0.366	0.653
Palliative	43 (59.7%)		
Primary	18 (25.0%)		
Bone-Targeted Therapy			

Var□able & Subgroups	n (%)	OS (p-value)	PFS (p-value)
None	25 (34.7%)	0.910	0.971
Zoledronic acid	27 (37.5%)		
Denosumab	20 (27.8%)		
**Laboratory Markers**			
LDH Level (U/L)			
< 225	52 (72.2%)	**0.050**	**<0.001**
≥ 225	18 (25.0%)		
ALP Level (U/L)			
< 130	44 (61.1%)	**<0.001**	**<0.001**
≥ 130	28 (38.9%)		
HALP Score Median			
< 31	35 (48.6%)	**0.015**	0.140
≥ 31	37 (51.4%)		
SII Median			
< 654	36 (50.0%)	0.590	0.883
≥ 654	36 (50.0%)		
PSA Response at 3 Months			
≥ 50% decline	47 (65.3%)	0.558	0.180
< 50% decline	13 (18.1%)		
TTN			
3 months	15 (20.8%)	**<0.001**<br>3-6:0,121	**<0.001**<br>**3-6:0,049**

Var□able & Subgroups	n (%)	OS (p-value)	PFS (p-value)
9 months	16 (22.2%)	3-9:0,094<br>**3-≥12:<0,001**<br>6-9:0,330<br>**6-≥12:<0,001**<br>**9-≥12:<0,001**	3-9:0,195<br>**3-≥12:<0,001**<br>6-9:0,776<br>**6-≥12:<0,001**<br>**9-≥12:<0,001**
9 months	6 (8.3%)		
≥12 months	15 (20.8%)		

Note: Statistical significance ($p < 0.05$) is indicated in bold.

Abbreviations: ALP, alkaline phosphatase (U/L, units per liter); CCI, Charlson Comorbidity Index; CI, confidence interval; ECOG, Eastern Cooperative Oncology Group performance status; HALP, hemoglobin, albumin, lymphocyte, and platelet index; LDH, lactate dehydrogenase (U/L, units per liter); M1a/b/c, TNM staging for distant metastasis (M1a: non-regional lymph nodes, M1b: bone, M1c: other visceral sites); n, number of patients; OS, overall survival; PFS, progression-free survival; PSA, prostate-specific antigen (ng/mL, nanograms per milliliter); RT, radiotherapy; SII, systemic immune-inflammation index; TTCR, time to metastatic castration-resistant prostate cancer; TTN, time to PSA nadir.

Supplementary Table S2: Candidate Variables Entered into the Initial Multivariable Cox Regression Models

	PFS	OS
Univariate Significance	Visceral Metastasis (absent-present)<br>Bone Metastasis Volume (≥ 5 or <5)<br>Metastatic Stage (M1a/b/c)<br>Prior Abiraterone Use<br>LDH Level (≥ 225 vs. < 225 U/L)<br>ALP Level (≥ 130 vs. < 130 U/L)	Visceral Metastasis (absent-present)<br>Bone Metastasis Volume (≥ 5 or <5)<br>Metastatic Stage (M1a/b/c)<br>Prior Abiraterone Use<br>LDH Level (≥ 225 vs. < 225 U/L)<br>ALP Level (≥ 130 vs. < 130 U/L)
Clinically Predefined Variables	Age (year)<br>CCI<br>Baseline Total PSA (ng/mL)<br>TTCR (months)<br>mCRPC-Enzalutamide Duration (months)<br>HALP Score<br>SII	Age (year)<br>CCI<br>Baseline Total PSA (ng/mL)<br>TTCR (months)<br>mCRPC-Enzalutamide Duration (months)<br>HALP Score<br>SII
Methodological Exclusions	Enzalutamide Timing<br>(first mCRPC or subsequent line)<br>TTN	Enzalutamide Timing<br>(first mCRPC or subsequent line)<br>TTN

Abbreviations: ALP, alkaline phosphatase (U/L, units per liter); CCI, Charlson Comorbidity Index; HALP, hemoglobin, albumin, lymphocyte, and platelet index; LDH, lactate dehydrogenase (U/L, units per liter); M1a/b/c, TNM staging for distant metastasis (M1a: non-regional lymph nodes, M1b: bone, M1c: other visceral sites); n, number of patients; OS, overall survival; PFS, progression-free survival; PSA, prostate-specific antigen (ng/mL,

nanograms per milliliter); SII, systemic immune-inflammation index; TTCR, time to metastatic castration-resistant prostate cancer; TTN, time to PSA nadir.

Supplementary Figure S1: Kaplan-Meier survival curves for patients with metastatic castration-resistant prostate cancer. (A) Progression-free survival (PFS) of the study population. (B) Overall survival (OS) of the study population

A.

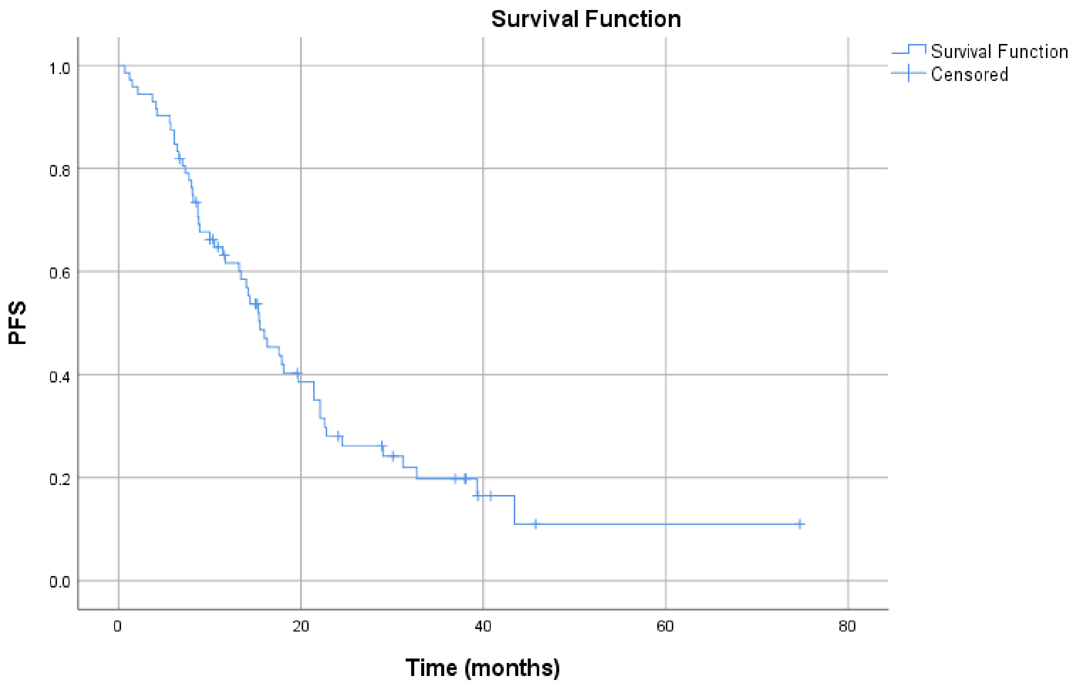

B.

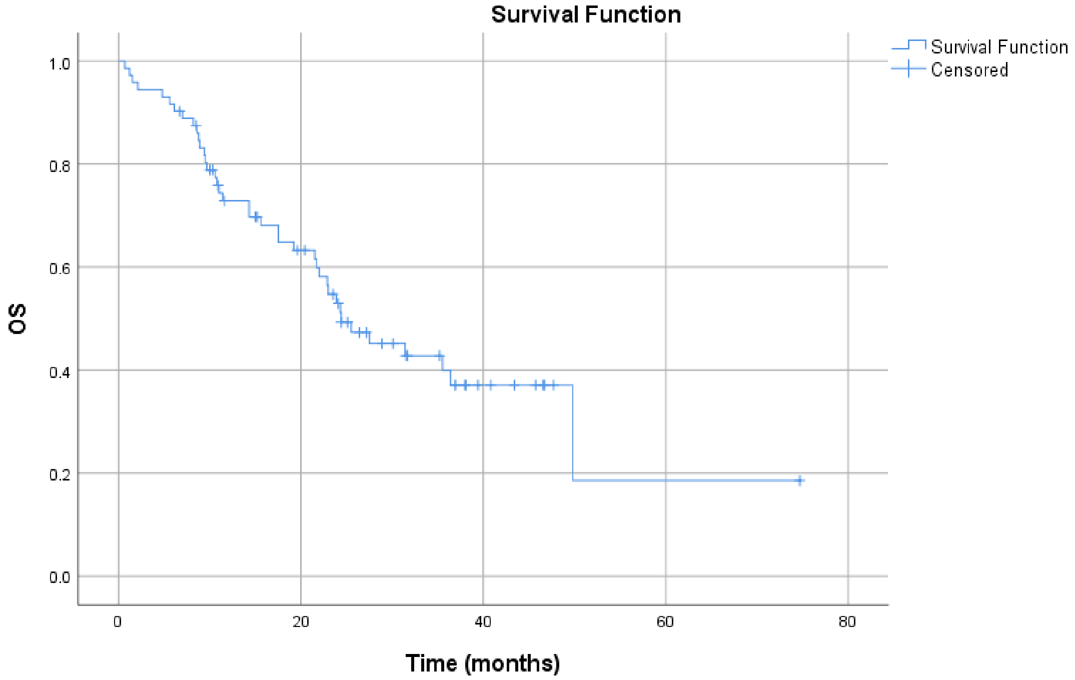

Supplementary Figure S2: Kaplan–Meier survival curves according to time to PSA nadir (TTN) categories

A.

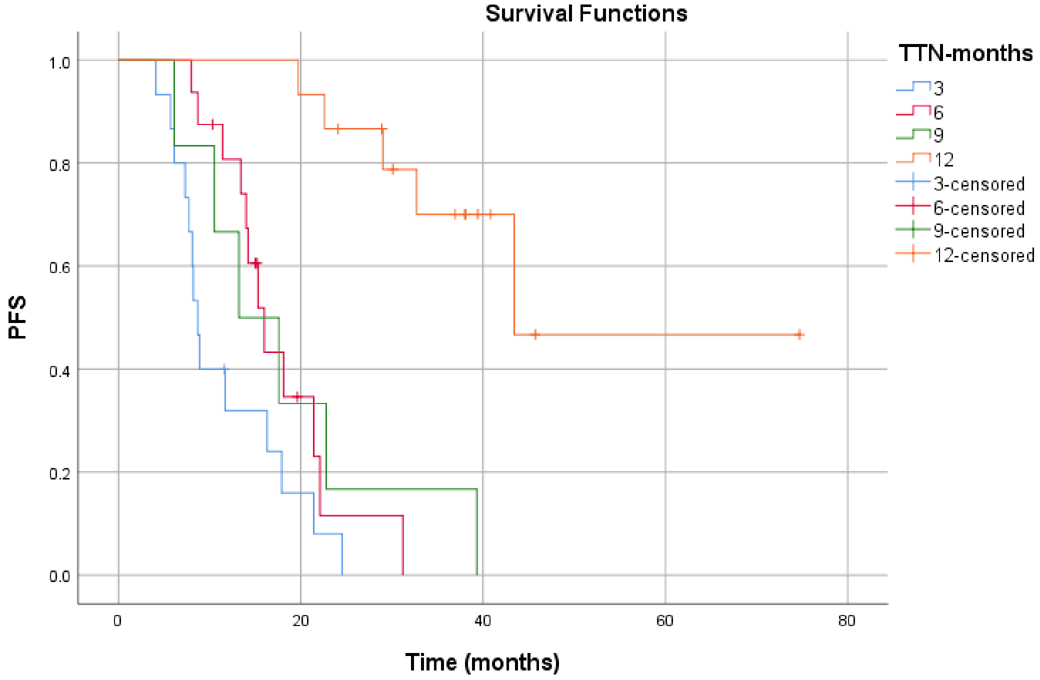

B.

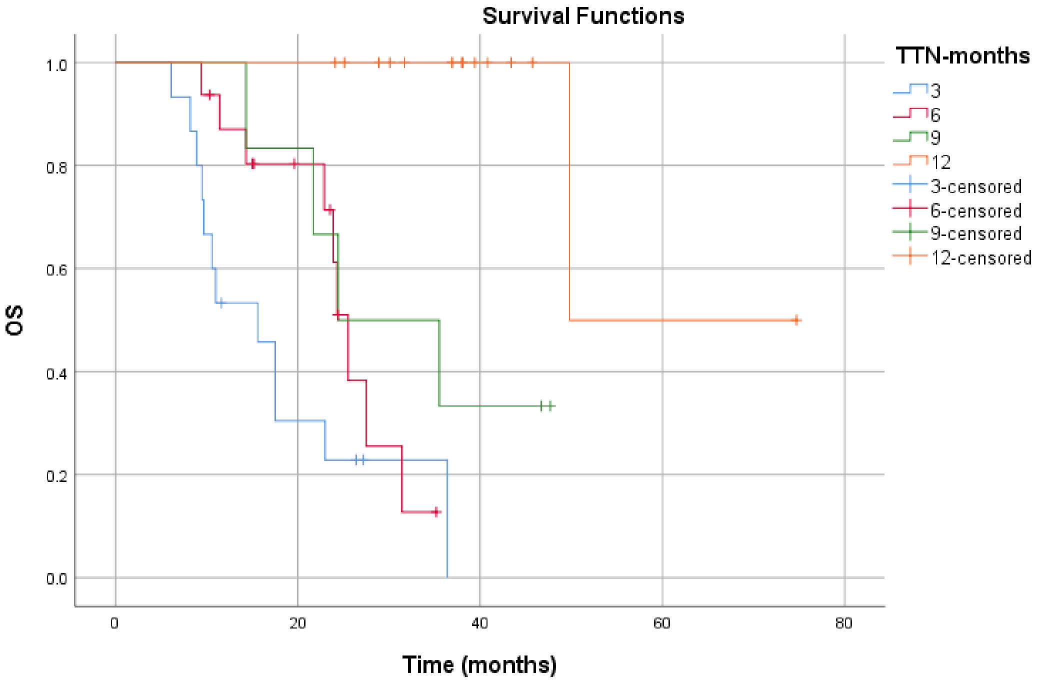

(A) Progression-free survival (PFS) stratified by TTN categories, showing a similar but less distinct pattern.

(B) Overall survival (OS) stratified by TTN (3, 6, 9, and ≥12 months). Patients with TTN ≥12 months exhibited substantially prolonged survival.

Tick marks represent censored patients.

Supplementary Figure S3: Forest plot of multivariable Cox proportional hazards models for progression-free survival (PFS) and overall survival (OS)

A.

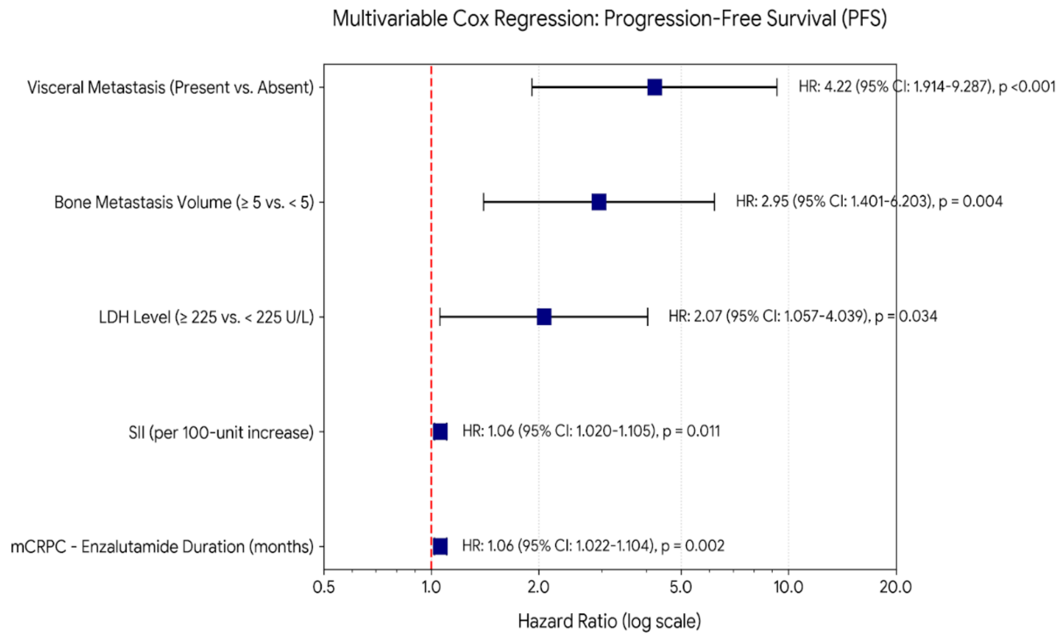

B.

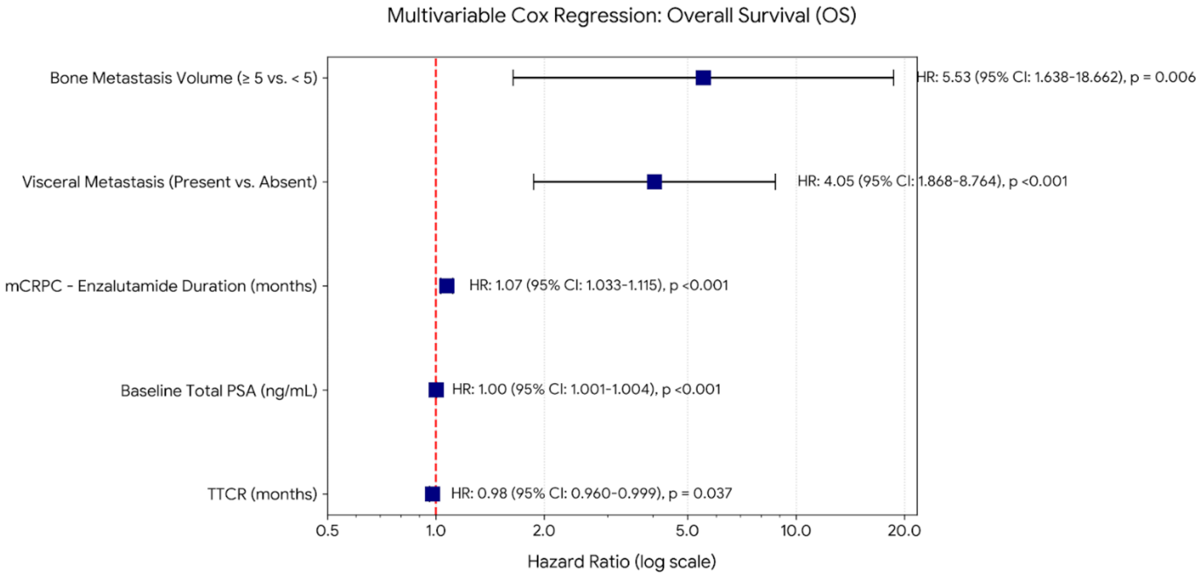

Abbreviations: CI, confidence interval; HR, hazard ratio; LDH, lactate dehydrogenase; mCRPC, metastatic castration-resistant prostate cancer; PSA, prostate-specific antigen; SII, systemic immune-inflammation index; TTCR, time to castration resistance.

Forest plot of multivariable Cox proportional hazards models for progression-free survival (PFS) and overall survival (OS). Hazard ratios (HR) with 95% confidence intervals (CI) are shown for each covariate retained in the final models. SII was scaled per 100-unit increase.
